# Xenodiagnosis to address key questions in visceral leishmaniasis control and elimination

**DOI:** 10.1371/journal.pntd.0008363

**Published:** 2020-08-13

**Authors:** Om Prakash Singh, Epco Hasker, Marleen Boelaert, David Sacks, Shyam Sundar

**Affiliations:** 1 Department of Biochemistry, Institute of Science, Banaras Hindu University, Varanasi, India; 2 Infectious Diseases Research Laboratory, Department of Medicine, Institute of Medical Sciences, Banaras Hindu University, Varanasi, India; 3 Department of Public Health, Institute of Tropical Medicine Antwerp, Belgium; 4 Laboratory of Parasitic Diseases, National Institute of Allergy and Infectious Diseases, National Institute of Health, Bethesda, Maryland, United States of America; University of Queensland, AUSTRALIA

## Abstract

Visceral leishmaniasis (VL) remains an important public health issue worldwide causing substantial morbidity and mortality. The Indian subcontinent accounted for up to 90% of the global VL burden in the past but made significant progress during recent years and is now moving towards elimination. However, to achieve and sustain elimination of VL, knowledge gaps on infection reservoirs and transmission need to be addressed urgently. Xenodiagnosis is the most direct way for testing the infectiousness of hosts to the vectors and can be used to investigate the dynamics and epidemiology of *Leishmania donovani* transmission. There are, however, several logistic and ethical issues with xenodiagnosis that need to be addressed before its application on human subjects. In the current Review, we discuss the critical knowledge gaps in VL transmission and the role of xenodiagnosis in disease transmission dynamics along with its technical challenges. Establishment of state of the art xenodiagnosis facilities is essential for the generation of much needed evidence in the VL elimination initiative.

## Introduction

**Fig 1:**
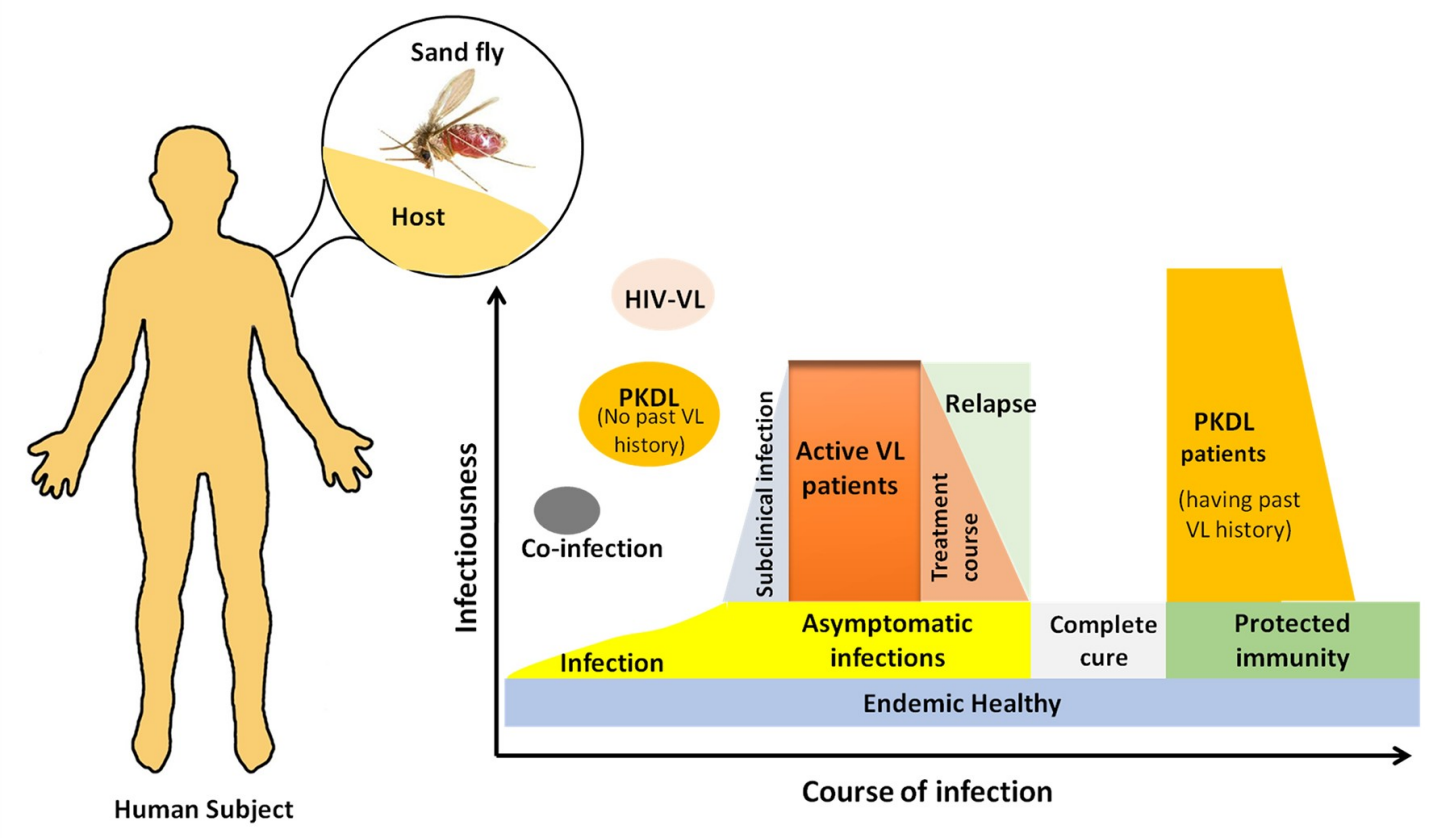
Dynamics of human infectious reservoirs for VL. Human infection is initiated when an infected female *P*. *argentipes* injects *L*. *donovani* parasites during a blood meal. Most infections do not lead to clinical symptoms and are suppressed or eliminated by the innate or adaptive immunity. Only a fraction of infected, asymptomatic subjects progress to clinical VL disease. After treatment of clinical infection, most patients develop protective immunity against the disease. Approximatly 1% to 10% of former VL patients may develop a chronic cutaneous form called PKDL. PKDL without history of VL is rare. HIV positive individuals are more at risk of developing VL once infected and become important source of transmission due to their potentially high parasitic load. PKDL, post kala-azar dermal leishmaniasis; VL, visceral leishmaniasis.

Neglected tropical diseases (NTD) have a disproportionate impact on indigenous, poor, and rural populations, accounting for important morbidity and mortality and major costs to healthcare budgets globally [1]. Leishmaniases, a group of vector-born diseases caused by at least 20 different species of *Leishmania* parasites, are among the NTDs with a huge impact in terms of disability-adjusted life years (DALYs) [2, 3].Visceral leishmaniasis (VL), also known as kala-azar, is the most severe form of leishmaniasis, with an estimated annual incidence of 0.2 to 0.4 million cases worldwide [2, 4]. The majority of VL cases are concentrated in just six countries, including India, Bangladesh, Brazil, Ethiopia, Sudan, and South Sudan [2]. Until recently, approximately 70% of those cases were reported from India, and the epicenter of infection is situated in the state of Bihar. In this region, the disease is commonly thought to have an anthroponotic transmission cycle, as no mammalian host other than humans has ever been shown to sustain transmission of the etiologic agent, *L*. *donovani* [5, 6]. In Europe and Latin America, to the contrary, the causative organism is *L*. *infantum*, which has the domestic dog as its main reservoir [7, 8]. On the Indian subcontinent, the parasite is transmitted by the female sand fly *Phlebotomous argentipes* [9]. Importantly, transmission of VL in India is very focal: It can be highly prevalent in some villages and nearly absent from others in the close vicinity. Within a particular village, the disease transmission is moreover highly clustered within certain hamlets [10, 11]. It has long been known that *Leishmania* infection does not always lead to overt clinical disease. Most infected humans remain asymptomatic, and only a small proportion (less than 10%) develop clinical symptoms. The incubation time period required by parasites to subvert host immunity varies, and depends on the environmental, parasite, and host-related factors (Fig 1).

VL is a fatal disease if left untreated [12, 13]. In 2000, it was estimated that over 75% of known kala-azar cases came from homes with a family income of less than US$1 a day [14] and could not afford their treatment, further exacerbating their poverty. Because of this fact, the morbidity and mortality of VL remained largely hidden and underestimated [15, 16]. After treatment and recovery of VL caused by *L*. *donovani*, some patients may develop a sequel with skin manifestations called post kala-azar dermal leishmaniasis (PKDL) that requires long and expensive treatment [17]. PKDL is not very common on the Indian subcontinent, with estimates of former VL patients affected ranging from approximately 1% to 10% [18, 19]. In East Africa, PKDL is a far more common complication, arising in up to 50% of people who have recovered from VL [20]. Although VL and therefore PKDL are due to *L*. *donovani* both in India and Sudan, Sudanese PKDL frequently self-heals (84% in 1 year), whereas Indian PKDL is thought to require more than one year for self-healing to occur [21]. PKDL patients represent an important but largely neglected reservoir of infection. It was shown that PKDL cases are infectious to sand flies [22], and they are thought to constitute an interepidemic reservoir [23].

VL had been virtually eliminated from India in the 1950s by intensive spraying of DDT during the National Malaria Eradication Program [24]. In early 1970s, a few years after DDT spraying had been stopped, there was a reappearance of VL cases in Bihar. The disease assumed a cyclical pattern, peaking at 77,102 cases in the year 1992 and at 32,803 cases in 2005. The disease also spread to neighboring countries, Bangladesh and Nepal. To counter this trend, a joint VL elimination campaign on the Indian subcontinent was launched in 2005 with the aim to eliminate VL as a public health problem by the year 2015. The deadline has since been extended to 2017 and, more recently, to 2020 [24]. The target was empirically defined as reduction of annual VL incidence to below 1 per 10,000 population at the health subdistrict level. The total reported VL case load in the region has diminished from over 50,000 in 2006 to fewer than 7,000 in 2016, with India reporting more than 90% of the latter cases [25]. However, the reduction in case notification rates fell short of the elimination target, in 2015 as well as in 2017, and even the extended deadline of 2020 will probably not be reached in India [4, 26, 27]. To date, Nepal has reached and maintained the elimination target to reduce the VL incidence below one case per 10,000 per year in each endemic district for the last three years; Bangladesh has achieved it in over 90% of its endemic subdistricts. [4] In India, the target was reached in 70% of subdistricts in 2016 and only 3128 VL cases reported in 2019, representing a 90% drop. Approximately 130 million people throughout the country currently remain at risk, and, as recently as 2019, India had more VL cases than any other country in the world. The failure of the Indian VL control program so far, despite very intensive efforts based on case detection and management as well as vector control, may indicate, besides programmatic issues, that active VL or PKDL patients may not be the only reservoirs of infection in endemic regions. On top of this, the disease has been reported from previously nonendemic areas [28]. The possibility that these patients were infected outside their home areas cannot a priori be excluded. However, given the increasing numbers of cases reported from nonendemic districts despite a lack of diagnostic tools and proper surveillance systems for VL in these districts, it would be careless not to consider the possibility of local transmission [29].

Theoretically, a single infected host may infect many sand flies for prolonged periods; therefore, infectiousness of the host for sand flies is a key parameter for determining the transmission dynamics of *L*. *donovani*, and mostly it depends on intrinsic and extrinsic factors such as severity of disease (i.e., parasite burden), host contact rate, host behavior, and host abundance [30]. Importantly, several studies on the Indian subcontinent have reported the prevalence of *L*. *donovani* infection in wild *P*. *argentipes* population ranging from 0.46% (Nepal) to 65% (Sri Lanka) and are listed in Table 1. These studies on sand fly infection in endemic areas provide some insight into VL transmission and raise two main questions to the scientific community and policymakers. First, is the observed decrease in reported VL cases attributable to the elimination efforts or is it due to a natural decline consistent with the cyclical pattern observed for VL in this region? Second, is it technically possible and operationally achievable to go one step further than “elimination as a public health problem” and really target “zero transmission” (i.e., eradication) in the long run? This would require monitoring of infection rates rather than clinical manifestations only and knowing more about which group of infected humans in endemic areas is serving as the principle reservoir for transmission of *L*. *donovani* to the sand fly vector. The possible reservoirs include active VL cases, individuals progressing to disease but not yet with overt symptoms, clinically cured cases, active PKDL patients, and infected but healthy asymptomatic individuals (**Fig 1)**. Several authors have suggested, based on mathematical modeling, that asymptomatic carriers of the parasites may also be infectious to sand flies [41]. However, this has never been empirically observed [41]. If asymptomatic carriers were shown to be infectious, this would cast doubt on the feasibility of driving the transmission of *L*. *donovani* down to zero, as there is no treatment available to eliminate the parasite from asymptomatic carriers [42]. Another question is whether the reservoir truly is strictly human, as some nonhuman vertebrates (e.g., domestic animals) were found positive for *L*. *donovani* parasites by polymerase chain reaction (PCR) [43, 44]. To inform the VL elimination policy it is thus important to quickly elucidate these lingering questions on latent parasite reservoirs [28, 45]. Importantly, now that the VL elimination program has achieved a significant reduction in incidence of VL in endemic areas, it has become even more important to sustain these reductions in transmission levels. Thus, the relative contribution of asymptomatic carriers may become more important in such context. The only way to decisively answer this question is via xenodiagnosis studies that evaluate and document the transmissibility of parasites from an infected host to the sand fly vector. Understanding the dynamics and epidemiology of anthroponotic transmission holds clear importance for the fine tuning of elimination strategies [28, 46]. In this policy-focused Review, we will discuss the role of xenodiagnosis in addressing the current knowledge gaps in VL transmission that threaten elimination and identify the operational research that can rapidly provide the missing information for timely incorporation into the VL elimination strategy.

**Table 1:** Studies on the Indian subcontinent estimating proportion of *Leishmania donovani–*infected *Phlebotomous argentipes* population.

S.N.	Study	Year	Region and Country	Assay	Infection prevalence in sand flies
1	Srinivasan et al. [31]	2016	Western Ghats, India	PCR	5.7% (5 of 88 pools)
2	Bhattarai et al. [32]	2009	Sunsari district, Nepal	PCR	0.578% (95% CI 0.218–1.11 (simulation method)), 0.486% (95% CI 0.0463–0.733 (Farrington method)
3	Pandey et al. [33]	2008	Dhanusa district, Nepal	PCR	6.7% (individual female flies)
4	Ostyn et al. [34]	2015	Okhaldhunga and Bhojpur districts, Nepal	PCR	14.5% (individual blood fed female sand fly)
5	Tiwary et al. [35]	2013	Muzaffarpur district, India	PCR	1.5% (0.85%–2.84%, depending on season)
6	Uranw et al. [36]	2013	Sunsari district, Nepal	PCR	10.74% (13 of 121 pools)
7	Gajapatrhy et al. [37]	2013	Hambantota, Sri Lanka	PCR	65% in randomly selected flies
8	Tiwary et al. [38]	2012	Muzaffarpur district, India	PCR	4.9%–17.37% (minimum to maximum, pooled analysis)
9	Kumar et al. [39]	2001	Vaishali, India	Microscopy	0.1%
10	Dinesh et al. [40]	2000	Vaishali, Muzaffarpur, and Patna districts, India	PCR	32% (12 of 38 pools)

### Xenodiagnosis

**Fig 2:**
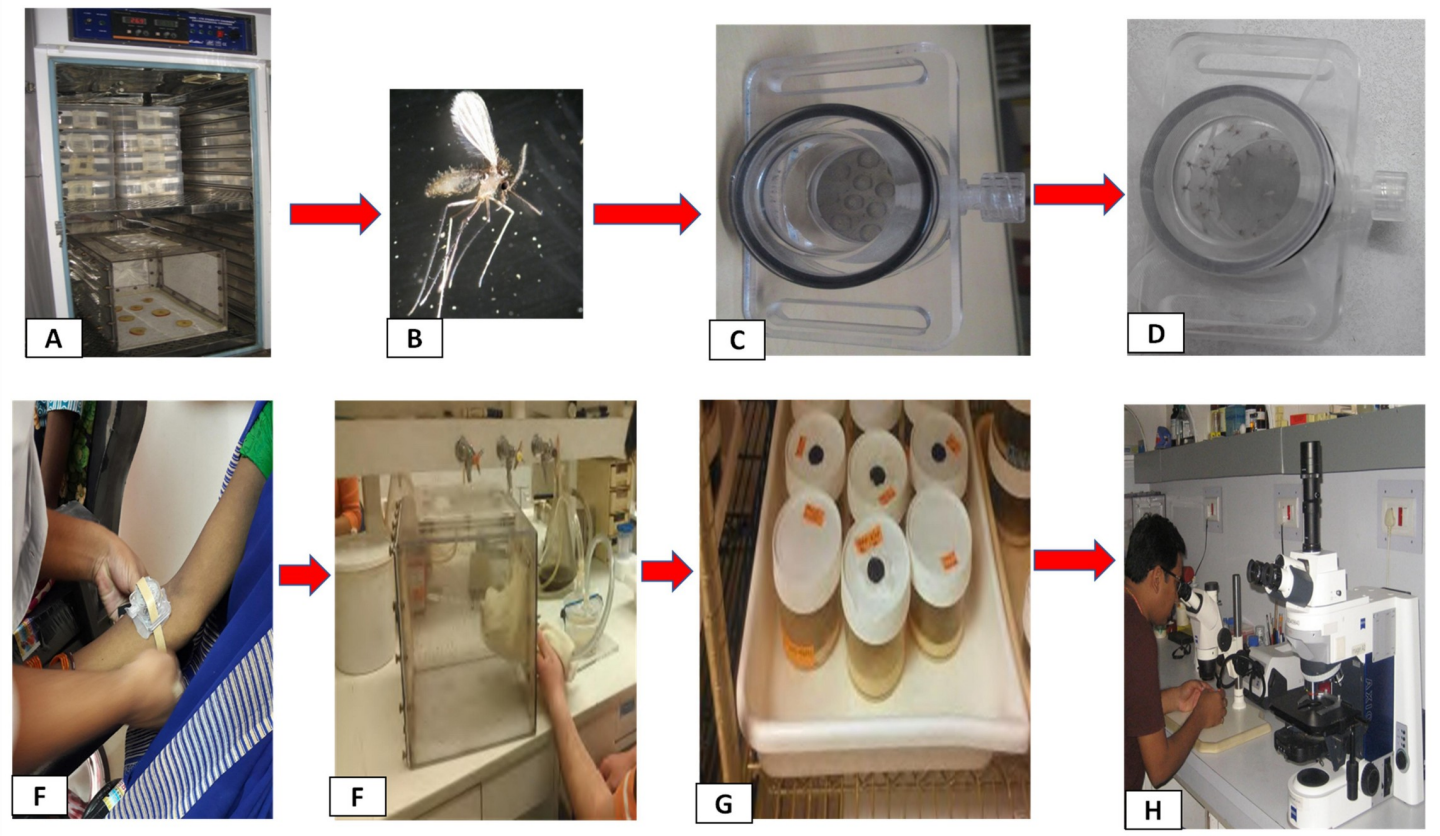
The infectivity of the subject can be tested with direct xenodiagnosis using sand fly vector female *P*. *argentipes*. **(A)** Pathogen free colony of sand fly. **(B)** Three-to-five day old unfed *P*. *argentipes* females removed from a colony cage. **(C)** An empty 2-inch diameter polycarbonate feeding chamber. (**D)** Loaded with sand flies by mouth aspirator through a small entry port. The feeding chamber has a screen-mesh bottom, through which the sand flies can feed, and vented top to prevent moisture condensation that might entrap sand flies inside the chamber. (**E)** The loaded feeding chamber (with 20 to 30 female *P*. *argentipes*) is then strapped with the screened side against the skin by a band for 30 minutes on the arm or leg (location can be decided during xenodiagnosis process based on logistic as well as patients comfort). This procedure usually takes place in the xenodiagnosis room, separate from the closed colony insectary room. **(F)** After 30 minutes of feeding, the feeding chamber is removed from the patient’s arm or leg and sand flies are released from the feeding chamber into a polycarbonate holding cage where they can be separated as fed and unfed. **(G)** Only blood-engorged females sand flies are transferred into a 1-pint holding container with screen-mesh top and held for at least 72 hours in an environmental cabinet at 28°C and 80% humidity with a 17:7 (Light: Dark) photoperiod. **(H)** All 3 to 5 days postfeeding surviving flies are then dissected and examined under the microscope for the presence or absence of *Leishmania* promastigotes. A subject will be considered positive for infectivity to sand flies if an infection is observed in at least one sand fly.

Xenodiagnosis is a diagnostic procedure in which the insect vector is used as a culture medium for the detection of infection in a mammalian host [47]. The method is also used in an experimental set-up to measure the proportion of insects that become infected after feeding on known infected hosts and allows studying their infectiousness. This method was introduced in the early 20th century as a way of detecting trypanosomes in mammalian hosts by feeding laboratory-bred reduviid bugs on the animals [48, 49]. Xenodiagnosis was (and is still sometimes) used in clinical medicine for the diagnosis of *Trypanosoma cruzi* infection in Chagas disease with good sensitivity when combined with a PCR [50]. Similarly, when used as a tool to confirm the diagnosis of seropositive patients infected with arboviral infections, xenodiagnosis has been shown to be both specific and sensitive (reviewed in ref.[49, 51]). Xenodiagnosis can be performed in two ways: In direct xenodiagnosis, live insects are used to detect viable disease organisms in individuals with presumptive infections **(Fig 2)**. This is the method that has been historically employed for the diagnosis of American trypanosomiasis caused by *Trypanosoma cruzi*, where the Triatoma bug is used [52]. This technique involves the feeding of noninfected hematophagous insects on a suspected infected individual. After incubation and amplification in the body of the insect, the parasites, if present, may be easily visualized and recovered from the digestive tract. Indirect xenodiagnosis on the other hand, refers to insect feeds on heparinized blood through a feeder membrane (i.e., chicken skin). This method not only avoids the risks posed by direct xenodiagnosis, mainly hypersensitivity to insect bites and transmission of other infectious agents, but also increases the proportion of sand flies that actually feed on the blood [53].

Thus, xenodiagnosis is the classical parasitological method to determine and quantify the transmissibility from a specific host to an insect species and provides conclusive data to discern infectious from noninfectious hosts. Hence, we postulate it can be applied to investigate the characteristics of *L*. *donovani* transmission in an endemic area and provide the epidemiological and clinical evidence required to guide VL control programs. Such information has multiple practical implications, including: (1) determining whether the current VL drug treatment in India affects the reservoir potential of the human host; (2) assessing the need for development of more efficacious treatments for PKDL (or strategies for decreasing the infectiousness of those with PKDL); (3) the potential role for prophylactic therapy for incident asymptomatic infection; (4) the potential role of active case finding in order to decrease the interval between the onset of symptoms and treatment; (5) the value of a proposition for the development of a vaccine that could prevent infection or infectiousness; (6) the required duration of the vector control program.

In the following section, we will discuss the use of xenodiagnosis in investigating the importance of antroponotic versus zoonotic mode of disease transmission, in understanding the role of asymptomatic infection in disease transmission, understanding the role of PKDL in *Leishmania* parasite transmission, and measuring treatment and vaccine efficacy.

### Anthroponotic versus zoonotic transmission: an unresolved mystery on the Indian subcontinent

On the Indian subcontinent, *L*. *donovani* infection has long been considered an anthroponotic disease [46]. This is supported by the studies indicating that high levels of infection occur within populations and indoor dwelling of sand flies with these infected people led to continued infection [54]. However, it is still not clear whether all VL transmission on the Indian subcontinent is occurring in indoor settings or if outdoor biting *P*. *argentipes* are also contributing to incidence. India currently has intensive vector control programs (indoor residual spraying [IRS]) for VL elimination with the aim of reducing indoor vector densities of *P*. *argentipes* to a level at which VL transmission is reduced or eliminated [41, 55]. Importantly, it has been reported that environmental factors acting on the *Phlebotomus* vector (e.g., DDT spraying inside the house) can alter exposure of the human host to infected sand fly bites [56]. Recently, it was observed that the behavior of *P*. *argentipes* may have changed from endophilic (indoor resting) to exophilic (outdoor resting) as a consequence of IRS, [28, 57, 58]. This may have resulted in adapting to other sources of blood meals, including cattle, goats, water buffalo, dogs, and rodents, found predominantly outdoors [59]. These observations allude that one or more of these species of nonhuman vertebrates may emerge as reservoirs. Studies in canine leishmaniasis, particularly in South America and Europe have confirmed that dogs have detectable *L*. *infantum* parasites that can be found in sand flies in xenodiagnosis and, thus, provide a sufficient evidence of reservoir to maintain endemic parasite cycle in these countries [60–62]. To date, it is not known to what extent these nonhuman vertebrates can serve as a reservoir host to *L*. *donovani* on the Indian subcontinent and whether they can actually sustain *Leishmania* transmission. But, if confirmed, it would require a major change in the VL elimination policy. Xenodiagnosis can be applied to investigate the role of nonhuman reservoirs in the shifting ecology of *L*. *donovani* transmission. Importantly, recent studies on the potential impact of sand fly feeding on other domestic species in India and Nepal found PCR evidence of infection in nonhuman vertebrates (cattle, water buffalo, and goats) [43, 63]. Similarly, *Leishmania* DNA was detected in one stray dog from a VL-endemic area of Bangladesh [64]. Furthermore, there is a report of *L*. *donovani* infection in symptomatic dogs in Sudan [65]. However, mere positive result of PCR test in the blood of nonhuman vertebrates is not enough evidence to confirm their infectiousness to sand flies as these nonhuman vertebrates may just turn out to be dead-end hosts [30]. Infectiousness of these nonhuman vertebrates urgently needs to be explored because, if transmissibility were to be confirmed by xenodiagnosis, this would again present a formidable challenge to VL control programs.

### Resolving the long-standing debate on the role of asymptomatic infection in disease transmission

Infections due to *Leishmania* are often asymptomatic (**Fig 1**) [66]. Such asymptomatic subjects are in good health and, thus, never targeted for treatment as current antileishmanial drugs are too toxic [67, 68]. The ratio of incident asymptomatic infections to clinical cases in large population studies has been shown to be 4 to 1 in Bangladesh [69] and 8.9 to 1 in India and Nepal [70]. The exact incubation period for VL varies but is estimated to be several months [67, 70]. Since asymptomatically infected subjects greatly outnumber clinical VL cases they may play a major role in transmission, even if less infectious than clinical cases [42, 68]. It is assumed that most infections spontaneously resolve, but until resolution occurs, they may be a reservoir of infection to sand flies in the community [42, 68]. Some observational and modeling studies are supporting this hypothesis [41, 46] Importantly, one of the most challenging aspects of studying asymptomatically infected subjects is that they are not uniformly defined. To assess the potential role of asymptomatically infected persons in transmission of VL, we first need to be able to accurately identify these individuals and assess the extent to which they are infectious. Since the tests used for diagnosing VL infection have not been validated for this purpose in asymptomatic individuals, we need to better study the characteristics of these tests in this particular group. A specific test for *Leishmania* infection could be used to answer some of these fundamental questions in VL epidemiology and also to plan future requirements for VL control programs, including targeted vaccination programs once an effective vaccine becomes available.

At present, however, there are no validated tools available for monitoring of infection. Serological tests (rK39 dipstick test, rK39 ELISA, and/or direct agglutination test) routinely used for diagnosis of VL have been validated to detect disease but not to detect infection [71]. From several prospective studies, we know that vast majority of healthy people with a positive VL serology never progress to disease [66, 70, 72]. It is still not clear whether those seropositive healthy individuals in endemic areas were truly infected with parasites or whether the serology test results were just false-positive results due to cross reactivity or a manifestation of a prior infection that has been cleared. A recent study does show a substantial decline in seropositivity rates in Nepal, consistent with an observed decline in incidence of the disease [73]. Furthermore, *Leishmania* DNA has been detected by PCR in peripheral blood of persons with asymptomatic infection in India, Nepal, Italy and Brazil, [66, 74–76]. However, these population subgroups (positive serological test and/or positive for cellular immunity and/or presence of parasite DNA by PCR) do not overlap entirely and thus probably have varying degree of infectiousness [66]. Le Fichoux and colleagues cultured promastigotes of *L*. *infantum* from the buffy coat of nine out of 76 asymptomatically infected blood donors in southern France. [77]. These findings confirm the presence of live parasites in asymptomatic hosts, but they are not conclusive with regards to their infectiousness potential to sand flies, and thus proper xenodiagnosis experiments are crucial in this regard. We still do not know if asymptomatic subjects are infectious to sand flies, and this piece of evidence is crucially lacking to guide VL elimination policy. At least in the case of canine VL due to *L*. *infantum*, it is known that asymptomatic infections can be highly transmissible to sand flies; in one study, 93% of asymptomatic dogs versus 67% of symptomatic dogs were able to infect sand flies [78]. In East Africa where *L*. *donovani* infection is transmitted by *P*. *orientalis*, the lowest infection dose (2x10^3^ per ml of rabbit blood) was sufficient for the successful establishment of infections in about 50% of the sand flies by membrane feeding [79]. Mathematical modeling of cohort data obtained in north Ethiopia suggested that among the 14% infected persons (kinetoplast DNA [kDNA] specific quantitative PCR [qPCR] positive) in the population, those with the highest parasitemia (3.2%) were responsible for 62% of the infections in sand flies [80].

A study involving xenodiagnosis of human VL caused by *L*. *infantum* reported successful transmissions to sand flies by six out of 6 VL–HIV coinfected patients in Spain [81] and 11 out of 44 VL patients in Brazil [82]. However, the latter study failed to detect any infections in sand flies allowed to feed on 147 ‘asymptomatic’ subjects (22 ex- VL cases, 27 LST-positive asymptomatically infected and 98 contacts of human VL cases). In an experiment with direct xenodiagnosis of human VL in India, 1 of 183 *P*. *argentipes* sand flies allowed to feed on VL patients during the day time versus 4 of 75 fed around midnight were infected with *L*. *donovani* [83], suggesting that there may be a periodicity to blood or tissue parasitemia. Therefore, a carefully designed xenodiagnosis study on asymptomatic subjects in the Indian subcontinent can evaluate the reservoir competence of such infections and seems, as such, a crucial step to obtain much needed evidence for the VL elimination policy.

### Resolve the mystery of PKDL

PKDL is a clinically overt manifestation of parasite persistence in clinically cured VL patients, and their presumed role in disease transmission has been emphasized by the high rates of sand fly infections following exposure of the sand flies to the nodular PKDL lesions [23, 84]. It is thought that PKDL patients may play an important role in the transmission of VL, and chronic PKDL patients have been implicated in major VL outbreaks in the past [23]. On the Indian subcontinent, the incidence of PKDL is 10% to 20% [85], and, when it occurs, it often does so many years after the acute infection. Most PKDL patients do not have other associated signs or symptoms, and, apart from the skin lesions, they are generally healthy. Hence, they are not inclined to seek treatment and may serve as silent reservoir for sustained disease transmission in the community. For PKDL, not enough is known about the prevalence of different forms of PKDL on the Indian subcontinent, and even less is known about their relative importance regarding infectiousness to sand flies.

Demonstration of parasites by microscopy in skin smear and biopsies from lesions and/or blood PCR positivity are used to confirm the diagnosis of PKDL [71]. Sensitivity of these methods is not satisfactory, especially with macular PKDL for which multiple sampling is required for diagnosis, raising ethical concerns associated with the risk of such an invasive procedure. To the contrary, xenodiagnosis on PKDL patients is considered noninvasive with minimal risk and ethically acceptable. In India, the first xenodiagnosis study was reported in 1933 on a single nodular PKDL patient that confirmed the infection in the *P*. *argentipes* sand fly [86]. Later on, a few more xenodiagnosis studies were conducted with a very limited number of PKDL patients, mostly in Northern West Bengal, to determine the possible cause of occasional outbreaks in this region [23, 87]. Molina and colleagues have recently conducted xenodiagnosis and found a correlation between sand fly infection rates and qPCR in skin and blood of PKDL patients in Bangladesh [22]. More recently, this group has investigated the relative infectiousness of VL and PKDL patients on larger cohorts in Bangladesh and found that 17 out of 21 nodular PKDL patients (81%) were able to transmit to sand flies versus 9 out of 35 macular PKDL patients (35%). Out of 15 VL cases included in the study, 10 (67%) were able to transmit [51]. There is now an urgent need to determine the prevalence and incidence of the different forms of PKDL (macular, maculo-nodular, or nodular) in India and confirm via xenodiagnosis their levels of infectiousness to sand flies.

### Measuring the treatment efficacy

Current VL treatment in India places substantial stress on both society and affected families. In the hyperendemic regions of India and adjoining areas of Nepal, the traditionally effective antileishmanial drug sodium stibogluconate (pentavalent antimony, Sb^V^) has rapidly lost its efficacy due to the development of drug resistance [6]. On the other hand, Sb^v^ is still effective and commonly used for VL treatment in Sudan [88]. Currently, a single dose of liposomal amphotericin B (AmBisome) is an effective VL treatment option and recommended as the first line treatment in the elimination program on the Indian subcontinent [89]. However, the high cost of the drug limits access in endemic areas and can lead to underdosing and incomplete treatment conducive to the emergence of drug resistant strains [90]. Importantly, it is currently not known if single dose liposomal amphotericin B treatment kills the parasites to a degree sufficient to stop transmission to sand flies or if clinically cured cases can continue to be infectious. Important in this respect is the fact that in nonhuman vertebrate studies, *L*. *infantum* infected dogs remained infectious after treatment [91–93]. It is also not known if alternative treatment are better at removing clinically cured individuals from the pool of infection reservoirs. For PKDL patients, the duration of treatment with oral Miltefosine is 12 weeks, and we still do not know about how long these PKDL patients are able to transmit infection to sand flies while on treatment or even after treatment. If the infectiousness of cured subjects is confirmed by xenodiagnosis, this would present another major challenge to the current VL control program, as there are few alternatives to replace the current drugs AmBisome and Miltefosine for VL and PKDL treatment, respectively.

### Development of novel methods to evaluate vaccine efficacy

Vaccination is the most straightforward option for sustaining reductions in transmission by eliciting long-lasting immune responses [94]. The development of a vaccine against human VL has been an ongoing effort for decades, but none are yet available for human use [95, 96]. A major problem in translating discoveries from animal models into a vaccine for humans is that strategies identified in experimental models do not work on humans. Research in this area is hampered by the unavailability of methods for measuring the vaccine efficacy using the natural infection environment. It has been observed in nonhuman vertebrate studies that vaccines that worked against needle challenge did not work against natural sand fly challenge [97, 98], suggesting that fly colonies that are established for xenodiagnostic studies might also be useful in evaluation of vaccines. There are clear ethical issues that would preclude the conduct of controlled trials of sand fly transmissions of *L*. *donovani* to humans, despite the fact that this is the gold standard method for measuring the efficacy of any future vaccine.

### Regulatory, ethical, and practical challenges with xenodiagnosis

There are several logistic issues with the establishment of xenodiagnosis that need to be properly addressed before its direct application to human subjects. Theoretically, xenodiagnosis implies the use of a sand fly vector to evaluate and document the transmissibility of a host, but the sensitivity and adverse effects, if any, of such methods are still unknown. Moreover, standard validated protocols, including which part(s) of the body to be exposed to sand fly bites, in xenodiagnosis, studies does not exists. Ethical issues have so far not fully been discussed, and bio-safety issues, such as the generation of a virus-free colony as well as the government authority to approve it, are lacking in many countries. Guidance specific to vector-based research is generally lacking in government guidelines and regulations. However, lack of specific safety regulations does not equate to the xenodiagnosis studies being unsafe; indeed, the risks and burdens for volunteers exposed to bites from certified pathogen‐free laboratory reared sand flies should be minimal and ethically permissible [51].

The sand fly colony to be used in xenodiagnosis should be maintained as a closed colony, meaning that no new sand flies should be infused into the colony from the field. Sand flies to be used on patients must have had no direct exposure to other animals, having been maintained on a sterile sugar solution or apple slices prior to being offered blood meals to patients. The sand fly colony must have been shown to be free of *Leishmania* and other protozoan parasites by dissecting and examining hundreds of randomly selected female sand flies representative of the colony populations.

Importantly, sand flies may transmit several other important human pathogens including *Bartonella bacilliformis* (the etiologic agent of human bartonellosis in Andean South America), the Sand fly fever viruses, Chandipura virus, and sundry other arboviruses [99]. It is therefore very important to rule out such arboviruses and ensure that colony is free of transovarially transmitted arboviruses associated with other human diseases so that it can be safely used for xenodiagnosis. Hence, representative random samples of female sand flies from the closed colonies must be used for screening of viral infection through PCR or other highly sensitive quantitative methods like RNA sequencing. This procedure must be repeated at certain intervals to ensure that the colonies remain free of arboviruses.

Most importantly, a thorough ethical review and informed consent procedure is needed, as there is no immediate benefit to the individual who accepts to participate in a xenodiagnosis study. However, the related risks are minimal so long as the research infrastructure complies with the above-mentioned criteria. Subjects from VL-endemic areas are in their homes exposed to sand flies are known to frequently bite in the areas as they are normally exposed and on a daily basis; thus, a sand fly bite feels much like a mosquito bite. Risks may include temporary discomfort, swelling, redness, warmth, or itching at the site of fly feeding [100]. These risks directly attributable to the study procedures are minimal, and arguably little greater than those faced by the subjects on a daily basis in a sand fly endemic region [101]. Importantly, communities in the endemic region have existing social divides (e.g., caste, religions, knowledge, etc.) that may be exacerbated by perceptions around diseases and infectivity status [102]. Therefore, problems might arise in asking members of the community to subject themselves to sand fly bites if they do not understand the connection to VL. Hence, robust community engagement should be used to preempt any such misunderstanding, while also helping the researchers to understand what the communities expectations are with respect to the xenodiagnosis study.

As a standard tool, xenodiagnosis is probably a more ethical method for sampling humans than basic blood sampling or skin lesion biopsy in the case of PKDL. On the other side, xenodiagnosis has some disadvantages that includes potential for some allergic reactions, requirement of laboratory infrastructure and long time period (2 to 5 days) to get the final results, limiting its utility for widespread use at population level, especially in resource limiting settings [103]. Its use in research is also limited due to difficulties in breeding and maintenance of sand flies in insectaries. Low blood meal feeding is a major issue in most of the xenodiagnosis studies [104]. Furthermore, microscopic examination of *L*. *donovani* infection in dissected sand flies is tedious, time consuming, and requires a well-trained entomologist. While positive xenodiagnosis is an undisputable proof of *L*. *donovani* infection in a suspected individual, a negative result does not rule out the presence of the parasite. This makes xenodiagnosis highly specific but potentially poorly sensitive (i.e., high false negative rates) and dependent on host as well as vector related factors, such as parasite load, and/or variation in implementation procedures, including number of sand flies used, feeding time, and survival rate of sand flies until the infection measurement [105]. In the most recent experience with direct xenodiagnoses of human VL in India, one of 183 *P*. *argentipes* sand flies fed during the daytime versus 4 of 75 fed around midnight were infected with *L. donovani [83]*, suggesting that there may be a periodicity to blood or tissue parasitemia, which would limit its field application. Importantly, xenodiagnosis has the disadvantage of representing less well the context in which a natural transmission occurs, and may not therefore reflect accurately the probability that parasites are picked up from the blood or skin during natural exposure to sand fly bites [30].

The earlier xenodiagnosis studies in India were conducted only on PKDL patients but not on VL and asymptomatic individuals. Furthermore, there is no literature available to confirm whether sand flies used in these studies were free from other pathogens. Recently, Kala-azar Medical Research Centre (KAMRC) in Muzaffarpur, India, and Surja Kanta Kala-azar Research Centre (SK KRC), Mymensingh Medical College, Bangladesh, have established working, closed, arbovirus free colonies of *P*. *argentipes*, originating from a stock of wild sand flies [106, 107]. Currently, these closed colonies are being used to carry out xenodiagnostic studies on human subject and nonhuman vertebrate groups. This is a promising development in the endemic regions, which will be instrumental in providing the direct clinical evidence to the government in order to make the policy decisions for sustaining the elimination and postelimination agenda.

Although xenodiagnosis has many advantages and presents a gold standard for testing of infectiousness, it has its limitations. First, the use of colonized flies that may not reflect the vector competence of field sand flies and, therefore, may over- or under-estimate transmission. Second, sand fly blood feeds are typically restricted to the extremities and the timing may not correspond to natural feeding patterns of sand flies. Thus, results may vary if there are differences in parasite levels between specific body areas and at different times of the day. Feeds on different body areas and during night-time hours could pose a technical limitation to determining actual contributions to transmission. While blood parasitemia might seem the most likely predictor of transmission success to flies, the fact that *L*. *donovani* can colonize the skin has left the relative contributions of blood versus skin an open question.

## Conclusion

For several decades, the usual methods of control of VL through vector control (indoor residual insecticide spraying), diagnosis, and treatment have resulted in a limited long-term impact on interruption of the transmission of the disease, significantly resulting in repeated resurgences and thousands of lives lost. The recent elimination initiative is seemingly making progress to reach the target, but the next five to ten years will be critical to ascertain that transmission is sustainably reduced and eventually halted. Xenodiagnosis has the potential to contribute meaningfully to the long-term implementation of the VL elimination effort on the Indian subcontinent by answering some of the fundamental questions in VL epidemiology and providing the scientific evidence about transmission potential of different human or animal reservoirs. Determining the rates of infection and infectiousness in sand flies will provide insight into the course of natural VL infections and could be utilized to improve models forecasting the development of VL epidemics.

### Key learning points

- Xenodiagnostic assays will contribute meaningfully to the long-term implementation of the VL elimination effort by identifying the relative contribution of human and nonhuman reservoirs that may be playing a greater role in the maintenance of the *L*. *donovani* transmission cycle in endemic areas.
- Xenodiagnosis tools are important in translational research using the natural infection environment.
- Such studies investigate the immunological events in the skin of VL and PKDL patients in the context of Phlebotomine sand fly bites and provide the natural environment to characterize the local immune environment in the skin in which parasites initiate infection and are transmitted back to sand fly vectors.

### Five key papers

1. Mondal D, Bern C, Ghosh D, Rashid M, Molina R, Chowdhury R, Nath R, Ghosh P, Chapman L, Alim A, Bilbe G, Alvar J. Quantifying the infectiousness of post-kala-azar dermal leishmaniasis towards sand flies. Clin Infect Dis. 2018 Oct 24. doi: 10.1093/ cid/ciy891. PMID: 30357373
2. Molina R, Ghosh D, Carrillo E, et al. Infectivity of Post-Kala-azar Dermal Leishmaniasis Patients to Sand Flies: Revisiting a Proof of Concept in the Context of the Kala-azar Elimination Program in the Indian Subcontinent. Clin Infect Dis 2017;65(1):150–3. PMID: 28520851.
3. Serafim TD, Coutinho-Abreu IV, Oliveira F, Meneses C, Kamhawi S, Valenzuela JG. Sequential blood meals promote Leishmania replication and reverse metacyclogenesis augmenting vector infectivity. Nat Microbiol. 2018 May;3(5):548–555. doi: 10.1038/s41564-018-0125-7. PMID: 29556108.
4. Valverde JG, Paun A, Inbar E, Romano A, Lewis M, Ghosh K, Sacks D. Increased Transmissibility of Leishmania donovani From the Mammalian Host to Vector Sand Flies After Multiple Exposures to Sand Fly Bites. J Infect Dis. 2017 Apr 15;215(8):1285–1293. doi: 10.1093/infdis/jix115. PMID: 28329329.
5. Courtenay O, Peters NC, Rogers ME, Bern C. Combining epidemiology with basic biology of sand flies, parasites, and hosts to inform leishmaniasis transmission dynamics and control. PLoS Pathog. 2017 Oct 19;13(10):e1006571. doi: 10.1371/journal.ppat.1006571. PMID: 29049371.
